# Facilitators and Barriers to Health-Seeking Behaviours among Filipino Migrants: Inductive Analysis to Inform Health Promotion

**DOI:** 10.1155/2015/506269

**Published:** 2015-08-25

**Authors:** D. Maneze, M. DiGiacomo, Y. Salamonson, J. Descallar, P. M. Davidson

**Affiliations:** ^1^South Western Sydney Local Health District, 59a Cumberland Road, Ingleburn, NSW 2565, Australia; ^2^Centre for Cardiovascular and Chronic Care, Faculty of Health, University of Technology, Sydney, P.O. Box 123, Broadway, NSW 2007, Australia; ^3^Centre for Applied Nursing Research, School of Nursing and Midwifery, University of Western Sydney, Locked Bag 1797, Penrith, NSW 2751, Australia; ^4^Ingham Institute for Applied Medical Research, 1 Campbell Street, Liverpool, NSW 2170, Australia; ^5^School of Nursing, John Hopkins University, 525 North Wolfe Street, Baltimore, MD 21205, USA

## Abstract

Understanding factors that influence health-seeking behaviour of migrants is necessary to intervene for behaviour change. This paper explores Filipino migrants' perceptions of facilitators and barriers to maintaining health in Australia. Open-ended survey item responses reflecting factors that assisted and hindered health following migration to Australia were inductively analysed. Three hundred and thirty-seven of the 552 survey respondents (61%) provided open-ended responses. Responses were grouped into two major categories: individual factors, including personal resources and cultural influences, and environmental factors encompassing both the physical conditions in the host country and health service access. Awareness of practices that enhance health was a major personal facilitator of health-seeking behaviour; however, competing priorities of daily living were perceived as barriers. Cultural beliefs and practices influenced health-seeking behaviour. Despite high self-rated English language skills in this population, new migrants and the elderly cited communication difficulties as barriers to accessing health services. Insight into facilitators and barriers to health-seeking behaviour in this less researched migrant population revealed tools for enhancing engagement in health promotion programs addressing healthy lifestyle.

## 1. Introduction

Health promotion practitioners struggle to develop strategies to encourage change in health-seeking behaviour that are effective and sustainable in reducing risk factors for lifestyle diseases. A reason for the difficulty may be the mismatch between individuals' and programs' priorities [1]. While health service providers emphasise the long-term benefits of behaviour change, participants may be more focused on the present personal costs of such change. In view of this, exploration of the facilitators and barriers to health-seeking behaviour of key populations may be useful in providing an essential information base for health promotion practitioners to address these personal costs. Tailoring health messages will likely facilitate uptake of planned interventions for the community.

It is recognised that migrants face several barriers in maintaining health in their adopted countries, particularly among those with inadequate host language skills. This has been reported as an important contributing factor to low levels of health-seeking behaviour (HSB) resulting in poorer health outcomes [2]. For the purposes of this study, HSB is broadly defined as actions undertaken to care for, maintain, and uphold one's health, regardless of current health status [3]. This includes, but is not limited to, general health-promoting practices, such as choosing healthy food options, engaging in regular physical activity, participating in health promotion and education programs, and accessing preventive health services, such as cancer screening. Little is known, however, about the HSB of Filipino migrants to Australia who, despite their non-English speaking backgrounds (NESB), often rate their functional English language skills (FELS) highly. FELS refer to threshold English language skills, defined by the Council of Europe [4] as the compilation of knowledge and skills required for basic, but effective, communication in a foreign environment. Contributing factors for the high FELS in this population group include a long history of Western colonisation resulting in the adoption of English as an official language in the Philippines, a high percentage of intercultural marriages with Australians, and a high level of formal education [5].

In Australia, the influx of migrants from the Philippines has steadily increased with each census year, similar to other developed countries. The most recent Australian census data (2011) showed that, among NESB immigrant groups, Filipino immigrants are the fifth largest in terms of population size and have one of the highest levels (95.5%) of self-rated English language proficiency [5]. They are often perceived as requiring less health service support compared to those with other NESB migrant groups who have observable English language difficulty [6]. Filipino migrants, despite having a high prevalence of Type 2 Diabetes [7], metabolic syndrome, and emergency presentations for diabetes complications [8, 9], have not received much attention in relation to health research and resource allocation, particularly in the Australian context. One study reported a 16.1% prevalence of diabetes among Filipino migrants in the US, markedly higher than their counterparts in the US (8%) [10] and Australia (7.4%) [11]. Moreover, studies among Asian American migrants show that Filipino Americans are more likely to be obese [12] and have higher rates of smoking in men and women [13]. These health issues are likely to escalate with longer duration of stay [9].

The high risk profile, coupled with a dearth of research in this population, prompts exploration of issues that affect HSB in Filipino migrants to inform strategies to address behaviour change in this group. The ecological framework proposed by McLeroy et al. [14] was used as a heuristic tool to guide this study. It postulates that health promotion models that integrate personal, social, and environmental influences affecting health allow for a broader understanding of the dynamic relationships of these factors on HSB [15]. Changes in the social environment are posited to have a domino effect in changing individual behaviours. Likewise, support from individuals can instigate a change in the social environment attesting to the reciprocal relationship between individuals and environment. This approach underscores the importance of social determinants of health and supportive public policies in the host countries in moderating HSB and access to health care among migrants [16].

## 2. Methods

### 2.1. Participants and Recruitment

This paper reports on open-ended responses that were part of a larger study that investigated acculturation and HSB of Filipino Australians. Purposive sampling and snowball methods were employed to recruit Australia-dwelling Filipino men and women from November 2010 to June 2011. Advertisements were disseminated in Filipino-specific print, web, and broadcast media, via social media (http://www.facebook.com/), and through recruitment at community events, church functions, and Filipino-specific community groups and associations. Participants were included if they self-identified as having Filipino heritage, were 18 years or older, and were currently living in Australia.

### 2.2. Instrumentation

The parent study involved a cross-sectional questionnaire that sought demographic characteristics and included validated questionnaires on acculturation, acculturative stress, health-seeking behaviour, religiosity, and physical and mental health status. Pilot testing of the 96-item questionnaire was undertaken with 20 participants. Data for this paper were taken from two open-ended questions that asked about barriers and facilitators to taking care of their health. Following pilot testing, wordings of the two open-ended questions were simplified to “*From your experience in Australia, what has been the most important factor that helped you in taking care of your health?*” (facilitators) and “*What has been the most important factor that has made it difficult for you to take care of your health?*” (barriers). These open-ended questions allowed respondents to put forward factors they deemed important without being constrained by a priori categories. In addition, this paper also reports on the results of the modified health behaviour questionnaire by C. R. Bausell and R. B. Bausell [3] which asked about smoking, alcohol intake, consumption of a high fibre diet, and exercise behaviours (the Cronbach's alpha of the 14-item scale used in this study was 0.82). Body mass index (BMI) was calculated by the researchers based on self-reported height and weight. Self-report of health screening participation, including mammograms and pap smears, was also included in this paper to demonstrate preventive health behaviour practices among Filipino migrants.

Participants were given the option of responding to a web-based survey using a secure online platform or completing a hardcopy questionnaire. Approval to undertake this study was obtained from the relevant university (H8617) and health service human research ethics committees (HREC13/LPOOL 29).

### 2.3. Analysis

All responses to the open-ended items were imported into Microsoft Excel 2010 for inductive analysis. Most responses were written in English, although some were written in* Tagalog* (a common dialect in the Philippines). One of the researchers, who identifies as Filipino and is fluent in Tagalog and English, translated these responses. Responses were initially classified into corresponding a priori categories “facilitators” and “barriers” [17]. These groupings were subcategorised following independent parallel coding [17] by three investigators (D. Maneze, M. DiGiacomo, and Y. Salamonson). Subcategories were guided by the ecological framework [14] which looked at individual resources and cultural factors, as well as environmental and health service access-related factors that responders deemed important in HSB. Responses that pertained to resources perceived by respondents to be personal were considered individual factors. Environmental factors included elements in Australia that were regarded by participants as conducive or detrimental to HSB such as the physical environment, its facilities, and sociocultural aspects. Culture in this study was defined as the beliefs, traditions, practices, and characteristics of people that have been inculcated over time which guide their perceptions and health behaviours [18]. Responses were only included in this category when it was deemed by researchers to be directly culture-related. Following independent coding, investigators (D. Maneze, M. DiGiacomo, and Y. Salamonson) met to discuss any discrepancies until consensus was reached. Responses were quantified and percentages of total responses were calculated for each category. Most of the respondents provided more than one response. Descriptive statistics were used to quantify responses.

## 3. Results

### 3.1. Characteristics of Participants

Of the 552 respondents to the larger questionnaire, 380 (69%) responded to one or both of the open-ended items.  Table 1 provides a demographic overview of respondents, who were mainly women with a mean age of 44 years and had been living in Australia for an average of 18 years. Participants reported a low rate of engagement in regular physical activity (34%) and conscious avoidance of fatty foods in their diet (48%). The prevalence of smoking was 33%, and, among women, participation in cervical and breast cancer screenings was 43% and 53%, respectively (Table 1).

### 3.2. Facilitators of HSB

**Figure 1:**
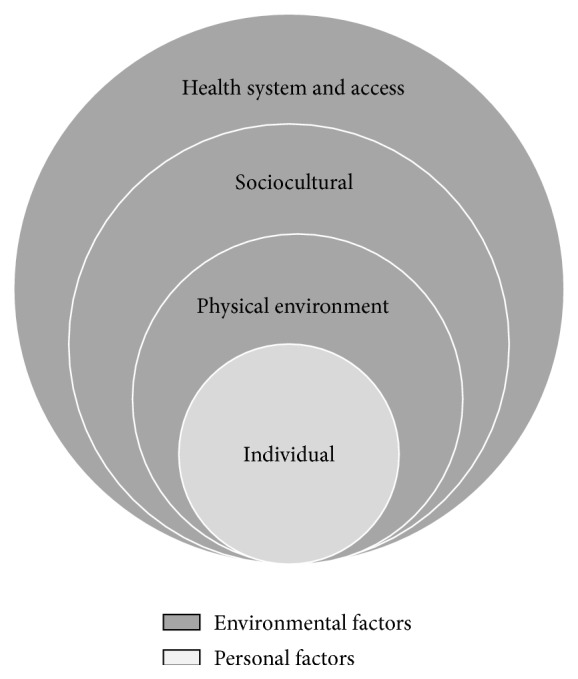
Ecological model for analysing HSB. Adapted from McLeroy et al. (1988) [14].

The study used the ecological framework [14] to categorise responses according to spheres of influence (Figure 1). Researcher-defined categories, percentages, and sample quotes are outlined in Table 2.

#### 3.2.1. Personal Factors

Two hundred and seventy-three responses (273) reflected personal factors that helped participants to take care of their health. These included having knowledge about good health, such as eating healthy, having a regular exercise regimen, and getting adequate rest and sleep, good social support networks, and being motivated to maintain health. Family was frequently mentioned as an important motivator in self-care and in making healthy lifestyle choices. In addition, some respondents were more aware of healthy behaviour because of a history of chronic disease among family members. Examples of these depictions were “Conscious of hereditary illnesses” (OL711) and “Heart problems in family's history. Don't want to become obese” (OL954).

#### 3.2.2. Cultural Factors

Spiritual faith in and intervention of the Divine Being were cited as facilitating factors in HSB for the deeply religious Filipinos. Use of traditional healing remedies commonly practised in the Philippines including herbal supplements and massage therapy was reported. The positive mental attitude of many Filipinos was written as a facilitator in taking care of one's health: “*Filipinos poor, but happy/joking/musical attitude, healthy*” (OL789).

#### 3.2.3. Environmental Factors

Participants wrote that the favourable physical environment in Australia was a contributing factor to general good health. “*Clean environment*” (OL923), “*peaceful environment*” (OL932), “*less pollution compared to the Philippines*” (OL619), and “*many parks, walking paths and leisure centres here*” (OL964) were reported facilitators of HSB. Sociocultural factors in Australia that were deemed to help in facilitating HSB included “*the Australian beach and sport culture*” (OL947) and “*seeing that there are a lot of obese people here and knowing the bad effects of obesity*” (OL745).

#### 3.2.4. Health Service Access and Policies

Having knowledge of and access to health services and health resources were important enablers of health care. Respondents also perceived the Australian health system as a facilitator of HSB. Examples included “*having an easily accessible health system*” (OL969), “*free health care*” (OL720),* “community health advertisements*” (HC 946), “*the Australian government's support*” (HC 960), “*medicare benefits*” (OL770, OL762, and HC990), and “*subsidised medicines for pensioners*” (OL696).

### 3.3. Barriers to HSB

#### 3.3.1. Individual Factors

The majority of responses (485) reported lack of personal resources, such as time and social support, and work-related factors such as job pressures, long working hours, and unemployment as barriers to HSB. Some respondents cited that physical impediments such as illness and disability and negative personal characteristics such as low motivation, laziness, and lack of self-discipline adversely affected their HSB. A lack of financial resources was similarly mentioned by a number of respondents as a barrier to HSB. Fifty-two responses (7%) stated no difficulty in HSB.

#### 3.3.2. English Language and Communication

Despite the high level of self-rated English language proficiency in this population, as evidenced by the 2011 ABS census report [5], some participants wrote about difficulty in communication and understanding Australian accents as personal barriers in using health services, particularly for newly arrived migrants and the elderly:

“*Always scared to see doctor coz it's hard for me to communicate; I don't know who or where the right person to talk to*” (HC903)

“*Language barrier (accent)*” (OL790)

“*… sometimes you can't express what you feel*” (HC911)

“*It's hard especially if you have difficulty in understanding the language*” (OL776)

#### 3.3.3. Cultural Factors

Perceived lack of consideration for cultural beliefs, traditions, and practices by health care professionals caused stress and was deemed a barrier to health service access. One of the respondents wrote the following under barrier to taking care of health in the host country: “*cultural beliefs that Australians do not understand. For example, the Australian nurse did not understand that I do not want to shower 30 minutes after delivery because it will make me sick*” (HC977).

Women with young children also reported that the lack of paid domestic helpers, a common cultural practice and source of support in the Philippines, as an additional challenge in finding time to take care of one's health, particularly for mothers and carers. While cultural gatherings helped foster cultural identity and increased social networks in the new country, they also encouraged consumption of large amounts of sugary and fatty cultural foods served during celebrations: “*Filipinos always have party and they cook so much food*” (OL843).

#### 3.3.4. Environmental Factors

Respondents described aspects of the physical environment as inhibiting HSB. Examples were the differences in climate and seasonal temperatures to which Filipinos were unaccustomed (OL802, OL889), “*dry air in Australia, not sweating like in the Philippines*” (OL799). Sociocultural influences acting as a barrier to HSB included the availability, affordability, and easy access to fast foods which was exemplified by the excerpt “*temptation of too much junk food around and it is always available*” (OL672, OL821). The perception of the Australian lifestyle as stressful and fast-paced compared to the Philippines prompted the quote “*Fast-paced life tends to make your forget about your responsibility of taking care of your body*” (OL643).

#### 3.3.5. Health Service Access

Barriers to HSB involved the lack of familiarity with the health care system in Australia, too few Filipino general practitioners (GPs), and the lack of information available in their original language. These barriers acted as impediments to accessing services, particularly for the new migrants and the elderly. The long waiting time to see specialists and the incompatible working hours of GPs made it difficult for workers to access this care, as depicted in the following excerpts: “*lack of GP access afterhours*” (OL931); “*can't get to see the GP or dentist on time because of shift work*” (OL921). Some respondents also felt rushed by the GP who seemed unsympathetic as in the following statement: “*GPs don't seem to listen to patient's symptoms and complaints. They seem to be in a hurry attending to patients*” (OL902).

## 4. Discussion

Findings of this study depicted Filipino migrants in Australia who were from a well-established community with an average duration of stay of 18 years. As in the 2011 ABS census data [5], respondents were predominantly female, highly educated and employed, and reported high levels of English language skills. Although these characteristics may suggest adequate capacity for self-care, a number of respondents had one or more chronic diseases and several risk factors such as being overweight and being a smoker. The prevalence of smoking in our sample was 33% which was much higher than the smoking rate of the general Australian population (18%) [19]. Breast (53%) and cervical cancer (43%) screening rates of Filipino women were below the Australian participation rates in 2010-2011 (58% and 57%, resp.) [20, 21].

Interestingly, despite the high rate of chronic conditions in the sample, many respondents rated their health as good or excellent. This is not dissimilar to the findings of Dela Cruz et al. [22] who found that Filipinos have good self-rated health despite higher than normal anthropometric measures, an indication of increased risk for chronic diseases. Becker [23] reported that a cultural characteristic of Filipino Americans is minimising the impact or presence of illness which could help explain this finding. Nevertheless, it cannot be discounted that it may also be an indication of a lack of comprehension about chronic disease, despite their high self-ratings in English language proficiency [24]. Understanding the meanings and implications of clinical management is different from the literal understanding of the language which if not taken into account could lead to miscommunication between providers and patients. This lack of awareness may have repercussions of less access to services for early diagnosis and risk reduction, self-management, and prevention of complications in chronic diseases.

### 4.1. Facilitators and Barriers to HSB

For many migrants who have settled in a new country in the quest of a better life, the practice of HSB are often perceived as a lower priority due to multiple and simultaneous settlement needs [6]. Many of the respondents wrote about practices that they believed promoted health, such as healthy eating and exercise, but there is ambiguity whether it is what they are actually practicing or whether these are behaviours they know they should be doing to maintain health.

Although facilitators and barriers to HSB in this study were categorised into individual and cultural, environmental, and health service access factors, the underlying cultural context of the responses was evident despite the small percentage of responses directly categorized as cultural factors. For example, individual factors such as lack of time and money were frequently cited by participants in this study as barriers to HSB. This finding affirms the precedence of fulfilling needs of daily living and establishing roots in the new country over health care, especially for new arrivals. Previous reports have shown that the impetus of most Filipinos to migrate is anchored on the cultural desire for improved socioeconomic positions for themselves and extended family living overseas [6, 25]. This may contribute to perceived economic pressures, thus compounding other financial concerns in the adopted country.

Employment was a personal factor that was a source of ambivalence, being identified as both an enabler and an impediment to HSB. Having a good command of English improved employment prospects in this migrant group [26], as well as being directly and indirectly correlated with HSB [27, 28]. For example, being employed increased financial capacity and social support networks, both facilitating HSB. However, scheduling health appointments within the working week was challenging given administrative requirements of taking leave of absence. Work responsibilities and stress were also deemed a hindrance to HSB by participants. A study among Filipino Americans confirmed that job-related stress was significantly associated with lower HSB and could lead to depression and development of chronic diseases [29].

Access to health services was a challenge for some participants. Australian health policies and the universal health care scheme facilitated subsidised general practitioner (GP) consultations and medicines for pensioners, yet having to engage with a different health system structure in Australia [30] and dealing with new terminologies and contexts can be confusing to new migrants and the elderly. For instance, the triage system in hospitals and after-hours care in Australia can be unfamiliar concepts that may hinder access for the unhabituated. In addition, despite the ability to speak English, some participants expressed a preference to seek health information written in their first language. The paucity of resources in* Tagalog* and the lack of Filipino GPs in their local area contributed to the barriers to HSB. Communication with health professionals was reported to be an issue for some participants due to difficulty understanding Australian accents [31] and being understood, two known barriers in intercultural communication [32]. For the elderly, cultural congruence and language concordance with their GPs have been found to contribute to better HSB via more comfortable and participatory consultations [33].

### 4.2. Cultural Factor as Facilitator and Barrier to HSB

In this study, cultural social networking was acknowledged to be an important resource in practising HSB as it provided social support and helped maintain and foster cultural identity [34]. However, it has also been reported to have detrimental effects on HSB. For example, foods served in cultural celebrations are usually high in fat and sugar making it difficult to follow dietary advice [23]. Health care providers may have difficulty in modifying diet and increasing physical activity among Filipinos because of the cultural significance attached to food. Among Filipino immigrants who had been living in the USA for an average of 15 years or more, changes in dietary practices showed higher consumption of a more Americanised diet including meat and fast food hamburgers and also increased salad and vegetables intake which was not traditional in the Filipino culture [22].

The rate of smoking and alcohol intake in this study could be a manifestation of establishing cultural networks and reinforcing the cultural value of* pakikisama* (camaraderie) as a means of coping with the stress of migration [35, 36]. “*Hiya*” is a cultural trait translated as “shame” or “a sense of propriety” [37] that may prevent Filipino patients from approaching health service providers for more information or clarification. Fear of offending health providers or being construed as challenging their expertise and authority may make Filipino patients more reticent and thus may affect HSB.

Being “time-poor” was frequently mentioned as a barrier in HSB in this study. This finding is consistent with Ko et al. [38] who identified a lack of time as a barrier to breast screening in Filipino American women. This was deemed to be a socially appropriate response among the Filipinos, conforming to the cultural practice of indirectly declining without causing offence. Alternatively, this is a reflection of the Filipino cultural trait of “*bahala na*” (loosely translated as submissive fatalism) which is a cultural belief that nothing can be done about the inevitable [39]. Nevertheless, it cannot be dismissed that this could also be due to a lack of awareness of the importance of screening. These meanings may not be understood if interpreted within the Australian or another Western cultural framework. A comparable low rate of breast and cervical screening in our sample could be a reflection of these cultural attitudes, cultural modesty, or even a fear of finding cancer, as in the report of Wu et al. [40].

Cultural meanings, attitudes, and beliefs impact behaviours. For example, a condition culturally termed “*pasma*” [41], which could be the cultural explanation for not showering after delivery cited above (*HC977*), is the belief that disease can be caused by an imbalance of “hot” state (pregnancy and delivery) and entry of “cold” into the body through the body pores believed to be opened up upon showering [42]. These cultural beliefs are hard to explain to health care professionals from different cultural orientations, language proficiencies, and authoritative status. Having a tolerant approach of the different cultural perspectives of illness causation may help to promote better patient-health provider relationship.

## 5. Implications of Research Findings to Health Promotion

Although Filipino migrants cited individual factors as highly important in HSB, many of these factors are underpinned by cultural beliefs, traditions, and practices in the home country. In addition, this migrant community may not have overt English language difficulties; however, this study showed that new arrivals and the elderly had difficulties in explaining their symptoms under stress of illness and in understanding the Australian accents and the terminologies used in an unfamiliar health care system. Colloquial English vocabulary differs from the lexis used in the clinical environment and therefore may necessitate additional support, despite having a level of English language skills. Furthermore, language proficiency statistics recorded in the ABS census data is a self-reported appraisal and not an objective measure. Health service providers should not assume that the ability to speak English equates having health literacy and competency in navigating the health system. It may be prudent to discreetly assess health literacy of nonnative English speaking migrants regardless of the level of English language skills.

Initiating culturally appropriate health promotion programs and designing effective health education tools to address the escalating health risks such as smoking and obesity in this population group are needed. These programs need to take into consideration competing priorities and support personal capabilities to empower the community to change behaviour. Examples of such initiatives may include web-based tools, workplace-based health programs, or flexible and weekend schedules of health promotion services to meet the needs of the high number of employed members of this community. Moreover, health promotion programs can also harness the strength of family support to initiate behaviour change. Cultural social gatherings, a common practice in the Filipino communities, can be used as platforms for health promoting information dissemination and program implementation.

More importantly, patient-held traditional beliefs will necessitate a deeper cultural understanding and open-mindedness among health workers. Cultural competency and intercultural communication trainings of health care providers have become more imperative in the face of increasing cultural and linguistic diversity of the patient population.

Further research is needed to build upon and elaborate on the findings of this study, as well as to explore additional environmental factors, such as health policies in host countries, that impact the HSB of Filipino migrants who are increasingly at risk of chronic health conditions.

## 6. Limitations and Strengths of the Study

The use of open-ended questions in this study enabled respondents to express their views without influence from the researchers. Yet, the meanings of responses were not always clear. For example, one-word responses could have been misinterpreted as it was difficult to ascertain the intended meaning and context of responses. To mitigate this limitation, data were coded and cross-checked by three investigators (D. Maneze, M. DiGiacomo, and Y. Salamonson). Filipino migrants living in rural Australia, those who are not participating in community events or organisations, or those who do not have internet access may have been inadvertently excluded from the study. A strength of this study was its focus on an underresearched population [6] whose health problems, behaviours, and health literacy may have gone unnoticed due to perceptions that they have adequate English language abilities or FELS. This study has highlighted the importance of questioning this potential misconception so as to improve the services, supports, and communication strategies for Filipino Australians.

## 7. Conclusion

This study demonstrates that Filipino migrants consider individual resources as important facilitators of HSB and the lack of these resources poses barriers. In spite of reporting on HSB facilitators in the adoptive country, English language proficiency, and familiarization with Western culture, Filipino migrants cite a number of individual, environmental, cultural, and access-related factors that hinder the practice of healthy behaviour. HSB needs to be understood within the cultural framework of migrants. Health promotion and other health care practitioners have to acknowledge that, despite FELS, Filipino immigrants are culturally diverse and, as such, cultural and traditional attitudes and beliefs may affect HSB.

## Figures and Tables

**Table 1:** Demographic and health characteristics of study participants (*n* = 552).

Characteristics	*n* (%)
Age, mean (SD) years, (range: 18–91)	44 (13.74)
Sex: female, *n* (%)	316 (67)
Country of birth: Philippines, *n* (%)	445 (95)
Duration of stay in Australia, mean (SD) years, (range: 0–42)	18.23 (9.5)
Educational attainment: tertiary level or higher, *n* (%)	363 (77)
Language spoken at home: speaks both Filipino and English, *n* (%)	383 (81)
In paid employment: yes, *n* (%)	365 (78)
Self-rated health: good or excellent, *n *(%)	292 (73)
One or more chronic disease(s): yes, *n* (%)	232 (42)
Body mass index (BMI): overweight or obese, *n* (%)	142 (35)
Smoker: yes, *n* (%)	174 (33)
Alcohol intake: more than 2 standard drinks a week, *n* (%)	65 (16)
High fibre diet, sometimes to never,* n* (%)	171 (42)
Exercise, sometimes to never,* n* (%)	278 (68)
Mammogram screening in female participants, 50 years or over, no, *n* (%)	58 (47)
Pap smear in female participants, 18 years or over, no, *n* (%)	125 (57)

^*^numbers in brackets are percentages except for age and duration of stay in Australian which are standard deviations.

**Table 2:** Analysis of themes in the written responses.

Themes	Definition and examples	Percentage of response	Exemplar comments
Facilitators		*N* = 505	

Individual factors	Health promoting behaviours, knowledge about health, current health needs or conditions, goals and motivation, and having the time and income and social support network	273 (54%)	*“Healthy eating habits, physical workouts, and God's blessings.”* (OL872, female, 53) *“Balance diet, exercise, rest, fun”* (HC988, female, 50)

Cultural factors	Spiritual beliefs in Divine assistance, alternative cultural therapies, positive cultural attitudes, and cultural beliefs and practices	50 (10%)	*“My belief that God will take care of me” *(HC958, male, 80) *“Filipinos poor but happy/joking/musical attitude” *(OL789, female, 39)

Environmental factors	Facilities available to support physical activity such as parks and walking tracks, availability of good quality and affordable food, and less pollution in the environment	106 (21%)	*“Seeking expert medical advice is not as expensive as that in the Philippines. More fresh fruit & vegetables for good nutrition within reach by average Australian” *(OL670, female, 56)

Health service access	Access to Medicare subsidised services such as GP consultations, GP quality, availability and accessibility of health resources, and supportive health policies	76 (15%)	*“Health information is readily available from various sources even if there is almost nil information written in Filipino or any of the Philippine languages”* (OL586, male, 43) *“Being able to have an easily accessible health system is the biggest factor in being able to take care of my health” *(OL969, Female, 28)

Barriers		*N* = 731^**∗**^	

Individual factors	Lack of motivation, negative personal characteristics (for example, laziness), lack of knowledge, lack of financial resources, and language difficulties Current health status that limits mobility, ageing, and unhealthy behaviours Competing priorities like work, family, and social commitments	485 (66%)	*“Money. dental care and eye care are expensive” (OL621, female,42)* *“I'm always scared to see a doctor because it's hard for me to communicate, I don't know who or where the right person to talk to” *(HC 903, female, 81) *“Balancing time taking care of the family and taking care of myself (taking time out to go to the doctor/physio/chiro/etc) is difficult”* (OL580, male, 30)

Cultural factors	Differences in cultural values, cultural norms and traditions, and difficulties in language expressions	86 (12%)	* “Looking after the family and working at the same time (the absence of domestic helpers specifically)”* (OL907, female, 42) *“By not understanding the life in this country. Australia people are different”* (HC977, female, 48)

Environmental factors	Environmental factors such as climate, lifestyle in Australia, and abundance of and easy access to fast food	72 (10%)	*“Junk food being readily available & so cheap.” *(OL786, female, 53) *“sudden change of weather/temperature”* (OL 802, female, 63)

Health service access	Lack of access to services such as after-hours GPs and lack of knowledge about the health system	36 (5%)	*“If you don't have relatives here in Australia, it is sometimes difficult to take care of our health especially you don't know how to access the health system and also if you have difficulty in understanding the language”* (OL776, female, 55)

^*^52 (7%) reported no barrier in taking care of their health.
